# Abdominal Aortic Aneurysms and Coronary Artery Disease in a Small Country with High Cardiovascular Burden

**DOI:** 10.1155/2014/825461

**Published:** 2014-02-20

**Authors:** Hassan Al-Thani, Ayman El-Menyar

**Affiliations:** ^1^Vascular Surgery, Hamad General Hospital, P.O. Box 3050, Doha, Qatar; ^2^General Surgery, Hamad General Hospital, Doha, Qatar; ^3^Clinical Research, Hamad General Hospital, HMC, P.O. Box 3050, Doha, Qatar; ^4^Clinical Medicine, Weill Cornell Medical School, P.O. Box 24144, Doha, Qatar; ^5^Cardiology Unit, Internal Medicine, Ahmed Maher Teaching Hospital, Cairo, Egypt

## Abstract

We aimed to evaluate the frequency, clinical profiles and outcomes of abdominal aortic aneurysms (AAA), and their association with coronary artery disease (CAD) in a small country with high cardiovascular burden. *Methods.* Data were collected for all adult patients who underwent abdominal computed tomography scans at Hamad General Hospital in Qatar between 2004 and 2008. *Results.* Out of 13,115 screened patients for various reasons, 61 patients (0.5%) had abdominal aneurysms. The majority of AAA patients were male (82%) with a mean age of 67 ± 12 years. The incidence of AAA substantially increased with age reaching up to 5% in patients >80 yrs. Hypertension was the most prevalent risk factor for AAA followed by smoking, dyslipidemia, renal impairment, and diabetes mellitus. CAD and peripheral arterial disease (PAD) were observed in 36% and 13% of AAA patients, respectively. There were no significant correlations between CAD or PAD and site and size of AAA. *Conclusion.* This is the largest study in our region that describes the epidemiology of AAA with concomitant CAD. As the mortality rate is quite high in this high risk population, routine screening for AAA in CAD patients and vice versa needs further studies for proper risk stratification.

## 1. Introduction

Screening programs for abdominal aortic aneurysm (AAA) are lacking in the developing countries. Global data reported that the prevalence of AAA varies in men (1.3%–8.9%) and women (11%–2.2%) [1].

The rising incidence of AAA is related mainly to the increase in age, physician awareness with clinical high index of suspicion, and the use of advanced diagnostic modalities. As AAA is a silent process, it may present only with aneurysmal rupture in most of cases. It was believed for a long time that atherosclerosis is the main pathogenesis of AAA. However, this speculation has raised a question that whether atherosclerosis is a “bystander” condition or an active factor for the initiation or acceleration of AAA [1, 2].

In a systematic review, Elkalioubie et al. evaluated 17 published studies between 1991 and 2010 [1]. The authors found that the frequency of AAA among coronary artery disease patients ranged between 0.48% and 18.2% [1]. As advanced age is a potential risk factor for both coronary artery disease and AAA, five studies specifically recruited patients who were above 60 years. Salem et al. [3] reported a lower prevalence of AAA among men of Asian origin (China and Iran), indicating that certain ethnic groups experience a disproportionately smaller burden of AAA. Notably, the prevalence of AAA in coronary artery disease patients was comparable to that reported in AAA screening studies among patients with different vascular bed affection such as peripheral vascular disease (PAD) (7%–17%) and cerebrovascular disease (8.4%–20.2%) [1, 4, 5]. Herein, we aim to evaluate the frequency, clinical profiles and outcomes of AAA, and its coronary artery disease association in Qatar. Qatar is a small rapidly developing country in the Middle East that is characterized with high prevalence of cardiovascular risk burden [6, 7].

## 2. Methods

Data were collected from March 2004 to March 2008 for all adult patients who underwent abdominal computed tomography scans at Hamad General Hospital in Qatar. All adult patients who underwent abdominal CT scans for any reason were included in the study and those with diagnosed abdominal aneurysms were followed up for 3 years for major events (AAA rupture and mortality). AAA was defined as an abnormal dilatation of the abdominal aorta with a maximal diameter of ≥3 cm. CAD was defined as history of documented coronary events, history of percutaneous or surgical coronary intervention or currently on anti-ischemic medications. PAD was defined as intermittent claudication, vascular lab diagnosis, or lower-limb arterial revascularization [7]. Data were reported in percentage, mean ± SD, and median and range, when applicable. Data analysis was carried out using the Statistical Package for Social Sciences version 18 (SPSS Inc. USA).

## 3. Results

**Figure 1:**
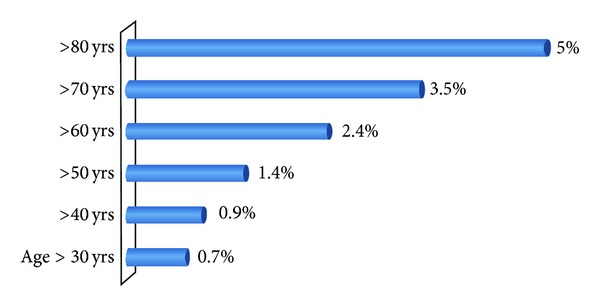
Abdominal aortic aneurysm in different age groups.

Out of  13,115 screened patients for various reasons, 61 patients (0.5%) were identified to have abdominal aneurysms. The majority of AAA patients were male (82%) with a mean age of 67 ± 12 years (Table 1). The incidence of AAA substantially increased with age reaching up to 5% in elderly patients (>80 years). The main location of AAA was infrarenal (67%), followed by thoracoabdominal (23%). The mean AAA diameter was 5.3 ± 2.5 cm with a range from 3 to 14 cm. AAA of diameter ≥5.5 and ≥7 cm comprised 41% and 26% of cases, respectively.  Figure 1 shows the AAA in different age groups.

The rate of AAA rupture was 8%, with an overall mortality of 33%. Among ruptured AAA, all patients were above 60 years old; 80% had AAA diameter of ≥5.5 cm and 60% of them died. Moreover, 80% of AAA located infrarenally.

Cardiovascular risk factors were prevalent among AAA patients in terms of hypertension (66%), smoking (60%), dyslipidemia (51%), renal impairment (46%), and diabetes mellitus (41%). CAD was prevalent in more than one-third of cases (36%), whereas PAD was observed in 13% of AAA patients. There were no significant correlations between CAD or PAD and site and size of AAA.

**Figure 2:**
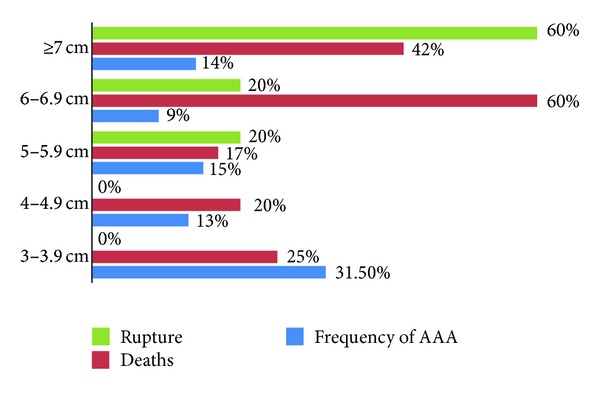
Rates of AAA rupture and deaths in different AAA size groups.

Figure 2 shows the rates of AAA rupture and deaths in different AAA size groups.

Table 2 shows the clinical profile of patients with AAA who had concomitant CAD. Patients with coexistent CAD were older (median age of 72 years); 91% of them were males and had high prevalence of cardiovascular risk factors as hypertension (86%), dyslipidemia (82%), and diabetes mellitus (54.5%). The mortality rate was 47% in patients with combined AAA and CAD, while AAA patients without CAD had 26% mortality rate.

## 4. Discussion

This is the largest study that describes the frequency of incidental AAA and concomitant CAD in the Arab Gulf Region. In our data, incidental abdominal aneurysms were detected in 0.5% of screened patients, of which the majority (67%) was ≥65 years old. The cardiovascular risk factors were prevalent among AAA patients in our study. Two-thirds of AAA patients were hypertensive, 60% were smokers, about half had dyslipidemia, and 41% had diabetes mellitus. History of CAD and PAD was observed in 36% and 13% of AAA patients, respectively. Among CAD patients, atherosclerosis risk factors such as age, smoking, and existence of other vascular bed affection showed an association with AAA [1]. This association addresses the need for screening for AAA in high risk population. Our analysis showed that the mortality was almost 2-fold greater in AAA patients if the history of CAD is present.  Table 3 summarizes the factors associated with increased risk of developing AAA and the recommendations for routine screening for AAA [8, 9].

It has been shown that not all patients with atherosclerotic disease develop an AAA. Few studies reported that many AAA patients lack concomitant atherosclerotic arterial disease [1]. Moreover, previous data did not correlate the existence or severity of each disease in the presence of the other one. Interestingly, this doubtful association presents in spite of the fact that AAA and atherosclerosis have several risk factors in common, such as male gender, increased age, smoking, and hypertension [10]. Some investigators believe that it would be useful to assess the relationship between the extent of coronary lesion and infrarenal aortic diameter and/or risk of AAA rupture [1]. On the other hand, some population-based studies showed that AAA is independently associated with preexisting CAD [11].

However, the high incidence of CAD among AAA patients was reported with an impact on the short-term survival post-AAA repair [12]. Studies that evaluated the coronary artery status prior to aortic surgery found a concomitant CAD prevalence from 31% to 90% [1, 11–13].

The retrospective nature of the current study is a considerable limitation. The breakdown and severity of CAD based on coronary angiographic features are lacking.

## 5. Conclusion

This is the largest study in our region that describes the epidemiology of abdominal aneurysm with concomitant CAD. The high prevalence of CAD does not express site, size, or severity of AAA so far. As the mortality rate is quite high in this high risk population, routine screening for AAA in CAD patients and vice versa needs further studies for proper risk stratification.

## Figures and Tables

**Table 1:** Clinical presentation and outcome of AAA.

	Number of patients	%
Total AA	61	0.5%
Age, median	69 (26–88)	
Age ≥ 65 yrs	41	67%
Infrarenal AAA	41	67
Thoracoabdominal	14	23
AAA rupture	5	8
Died	16	33
Male	50	82
Diabetics	25	41
Hypertension	40	66
Dyslipidemia	31	51
Smoking	37	60%
Coronary artery disease	22	36
Size of AAA	Mean 5.3 ± 2.5, median 4.8 (3–14)	
Size ≥ 5.5 cm	22	41%
Size ≥ 7 cm	14	26%

**Table 2:** AAA with history of coronary artery disease.

Age in yrs, median (range)	72 (44–82)
Male gender %	91
Diabetes mellitus %	54.5
Hypertension %	86.5
Dyslipidemia %	82
Peripheral arterial disease %	23
Age ≥ 60 yrs	91%
AAA location	
Infrarenal	68%
Thoracoabdominal	18%
AAA size (cm)	
≥5.5	30%
≥7	20%
Ruptured AAA	4.5%
Died	47%

**Table 3:** Factors associated with increased risk of developing an AAA.

Old age	
Gender	
Men develop AAA 4-5 times more often than women	
Ethnicity	
White people develop AAA more frequently than other ethnicities	
Vascular bed affection	
People with CAD and PAD are more likely to develop AAA than those who are otherwise healthy	
Family history	
A family history of AAA increases the risk of developing AAA	
The risk of developing an AAA among brothers of a patient with a known AAA who are >60 years old is as high as 18%	
Cardiovascular risk factors	
(i) Smoking: the risk is directly related to number of years smoking	
(ii) Diabetes mellitus: there is a negative association with diabetes mellitus and AAA	
(iii) Hypertension is a poor predictor for AAA development but important risk factor for expansion and rupture	
(iv) Lipid: there is no and weak correlation between risk for AAA and high serum triglyceride and cholesterol, respectively	
Recommendations for AAA screening	
Men of age 65–75 who have ever smoked should be screened one time for AAA with abdominal ultrasound	
Men > 75 are unlikely to benefit from screening	
Men age ≥ 60 who have a sibling or parent with an AAA should have a physical examination and abdominal ultrasound	
There is no recommendation for general screening for AAA in women	
(i) Women who have an increased risk for AAA (those who smoke have a family history of -AAA, or other risk factors) should be put into consideration	
(ii) The risk of rupture in women is higher than in men, and so some data are in favor of one-time screening for women with risk factors	
